# Antitoxin ε Reverses Toxin ζ-Facilitated Ampicillin Dormants

**DOI:** 10.3390/toxins12120801

**Published:** 2020-12-15

**Authors:** María Moreno-del Álamo, Chiara Marchisone, Juan C. Alonso

**Affiliations:** Department of Microbial Biotechnology, Centro Nacional de Biotecnología, CNB-CSIC, 3 Darwin Str., 28049 Madrid, Spain; mmoreno@cnb.csic.es (M.M.-d.Á.); cmarchisone@cnb.csic.es (C.M.)

**Keywords:** toxin-antitoxin system, cell wall inhibition, persistence, nucleotide hydrolysis, uridine diphosphate-*N*-acetylglucosamine

## Abstract

Toxin-antitoxin (TA) modules are ubiquitous in bacteria, but their biological importance in stress adaptation remains a matter of debate. The inactive ζ-ε_2_-ζ TA complex is composed of one labile ε_2_ antitoxin dimer flanked by two stable ζ toxin monomers. Free toxin ζ reduces the ATP and GTP levels, increases the (p)ppGpp and c-di-AMP pool, inactivates a fraction of uridine diphosphate-*N*-acetylglucosamine, and induces reversible dormancy. A small subpopulation, however, survives toxin action. Here, employing a genetic orthogonal control of ζ and ε levels, the fate of bacteriophage SPP1 infection was analyzed. Toxin ζ induces an active slow-growth state that halts SPP1 amplification, but it re-starts after antitoxin expression rather than promoting abortive infection. Toxin ζ-induced and toxin-facilitated ampicillin (Amp) dormants have been revisited. Transient toxin ζ expression causes a metabolic heterogeneity that induces toxin and Amp dormancy over a long window of time rather than cell persistence. Antitoxin ε expression, by reversing ζ activities, facilitates the exit of Amp-induced dormancy both in *rec*^+^ and *recA* cells. Our findings argue that an unexploited target to fight against antibiotic persistence is to disrupt toxin-antitoxin interactions.

## 1. Introduction

Bacteria have evolved complex regulatory controls and a diverse repertoire of cell transition states in response to various environmental stresses. In order to survive, cells slow down their growth rate and redirect their metabolic resources, until conditions improve and growth can increase [1]. It was shown early in *Staphylococcus aureus* that a large bacterial fraction is unable to survive to lethal doses of penicillin but a subpopulation survives, showing a biphasic inactivation curve [2,3]. It was proposed that in the presence of phenotypic heterogeneity (noise at the level of transcription of key genes during the life cycle), stochastic and antibiotic-induced stress leads to a non-genetic phenotypic “antibiotic insensitive” state, which was termed as persistence [3,4,5,6].Persisters have been featured throughout due to their important role in bacterial infections. In the Proteobacteria Phylum (*Escherichia coli* being the best-characterized representative) starvation-induced (p)ppGpp contributes to antibiotic persistence, whereas in bacteria of the Firmicutes phylum, the persistence effectors are more complex [7,8,9,10]. It has been proposed that in *S. aureus* the *bona fide* persistence effector is ATP depletion [11], but treatment with ATP synthesis inhibitors also induces (p)ppGpp accumulation and decreases GTP levels [12]. In *Bacillus subtilis*, which is the best-characterized representative of the Firmicutes phylum, the synthase-hydrolase RelA, and the two small alarmone synthases SasA (also termed RelP or YwaC) and SasB (RelQ or YjbM) downregulate ATP and GTP levels, increase (p)ppGpp, and indirectly the cyclic 3,5-diadenosine monophosphate (c-di-AMP) pools [13,14]. Antibiotic persistence might be controlled by deregulating (p)ppGpp and c-di-AMP pools [10,15]. In response to antibiotics that target cell wall biosynthesis, as the bactericidal β-lactam ampicillin (Amp), (p)ppGpp and c-di-AMP synthesis are induced in *B. subtilis* cells [16]. Furthermore, the *sasA* transcripts are increased by certain classes of cell-wall-active antibiotics (Amp among them) that trigger a membrane stress, and increase (p)ppGpp and c-di-AMP levels [13,17].

Bacterial toxin-antitoxin (TA) modules, which are ubiquitous across a broad range of extrachromosomal elements and are present in bacterial and archaeal chromosomes, are operons encoding a toxin that interferes with vital cell processes and an antitoxin that counteracts toxin action [18,19,20,21]. The type I to VI TA modules, which are classified according to the nature of the antitoxin and to their mode of toxin inhibition, are implicated in the fine tuning of multiple cellular functions and are associated with cell survival under different stress conditions [20,21]. The largest group is formed by type II TAs, which were first described as loci that enforce the maintenance of plasmids via post-segregational killing [22], and their ability to stabilize their mobile elements is considered as a selfish property [21,23]. In a type II TA system, which comprises a pair of genes coding for two proteins, the antitoxin forms a tight complex with the toxin to avoid its action [18,19,20,21,23]. Under certain stress conditions, the unstable antitoxin is rapidly degraded by host ATP-dependent proteases, freeing the proteolytically stable toxin [18,20]. The free toxin, which targets essential cellular processes such as RNA, DNA and protein synthesis, cell division, general stress response, etc., triggers a biphasic cell inactivation curve [24].

A variant of the HipAB type II TA system, rather than deletion of *hipBA* operon, has been shown to contribute to the formation of antibiotic persistence in *E. coli* cells. The *hipA*7 mutation, which maps in the HipA toxin, produces a high fraction of persisters [7,25]. HipA activates the stringent response, and by increasing (p)ppGpp levels, leads to persister formation [26,27,28]. However, a strong link between the induction of TA systems and antibiotic persistence is missing, and further work is needed to establish whether physiological levels of a toxin contribute to antibiotic persistence [23,29,30,31]. Deletion of *E. coli*- and *B. subtilis*-encoded chromosomal type II TA systems have no effect on antibiotic persistence [12,31].

The type II TAs of the ζ-ε superfamily, which is among one of the most broadly distributed TA modules in nature, are found in major human and plant pathogens. TAs of this superfamily are composed of two toxin monomers (with ζ and PezT as representatives), that interact with their cognate dimeric ε_2_/PezA_2_ antitoxin forming an inactive ζε_2_ζ or PezT-PezA_2_-PezT complex [32,33]. ζ/PezT interacts with its activator/substrate, the peptidoglycan precursor uridine diphosphate-*N*-acetylglucosamine (UNAG) [34]. UNAG is an essential precursor of bacterial cell wall biosynthesis [34]. The structures of the inactive ζε_2_ζ or PezT-PezA_2_-PezT complex, alone or UNAG bound, have been reported [32,33,34,35]. The interaction of the antitoxin with the toxin sterically blocks ATP-Mg^2+^ binding by the ζ/PezT toxin, but not with UNAG [32,33,34,35]. Therefore, the interactions with ATP and ε_2_/PezA_2_ are mutually exclusive [32,33]. The structure of the inactive ζε_2_ζ or PezT-PezA_2_-PezT complex, both alone or UNAG bound, has been reported [32,33,34,35]. Toxin ζ hydrolyses ATP in the presence of UNAG and phosphorylates a small fraction of UNAG, rendering it irreversibly inactive [15,34,36,37].

Transient toxin ζ expression, at or near physiological concentrations, leads to several cellular responses in *B. subtilis* cells growing in S7 minimal medium, at 37 °C with aeration. At early stages of transient toxin ζ expression (up to 15 min), the ζ UNAG-dependent ATPase decreases the ATP pool, and indirectly it alters the intracellular ATP/GTP ratios [37,38]. Such perturbation affects the concentration of the transcription initiation nucleoside triphosphates (ATP, GTP), and indirectly regulates RNA polymerase transcription initiation [39]. This mechanism, which is crucial for rapid adjustment of gene expression in response to environmental changes [40], alters the expression of ~2% of total *B. subtilis* genes (among those, it induces *relA* and *comGA* gene expression) [38]. However, an increased expression of SOS response genes or proteases that selectively induce cell-cycle arrest is not observed [38]. At 15–30 min times, the concerted action of *relA* and *comGA* increases the (p)ppGpp pool and indirectly the levels of the essential c-di-AMP second messenger [15,38]. Toxin ζ expression induces a cellular dormant state, but a reduced subpopulation, which shows no genetic modification, survives toxin action [10,15,38]. At 30–90 min, the synthesis of macromolecules (DNA, RNA, proteins) is inhibited and the membrane potential is impaired, with ~35% of total cells transiently stained with propidium iodide (an indicator of membrane compromised cells) [38]. At 90–120 min, the cell wall biosynthesis is reduced by ζ-mediated phosphorylation of a small UNAG fraction, leading to the accumulation of unreactive UNAG-3P [34,38]. In addition, (p)ppGpp mediates the inhibition of peptidoglycan metabolism [41]. Antitoxin ε expression blocks ATP binding by toxin ζ, thereby inhibiting its ATPase and phosphotransferase activities. Antitoxin ε reverses the toxic effect exerted by ζ in cell proliferation, and only ~10% of total cells remained stained with propidium iodide [38,42,43]. This is consistent with the fact that the toxin ζ action is reversible by nature even in distantly related bacteria, such as *E. coli* and *B. subtilis* cells [38,43]. Transient toxin ζ expression sensitizes cells to Amp treatment, and antitoxin ε expression reverses the ζ-induced dormant state, and the steady state condition is re-established [15,38,43]. The reversibility of ζ action upon longer periods of time in the presence of Amp is unknown. Therefore, we have re-examined the mode of action of toxin ζ, at or near physiological concentrations in *B. subtilis* cells growing in the minimal S7 medium, to understand how toxin ζ contributes to reduce Amp persistence.

In this study, we show that toxin ζ induces a dormant although still metabolically active state. The study of SPP1 in cells where toxin ζ is expressed reveals that ζ induces an active slow-growth rather than a growth-arrested condition and abortive infection. Amp addition at twice the minimum inhibitory concentration (2× MIC) halts *B. subtilis* proliferation, leading to a biphasic time-inactivation curve, which is maintained by a long period of time. Transient toxin ζ expression sensitizes cells to Amp action during a long time period. Antitoxin ε expression switches back ζ-induced dormancy, and promotes the exit of toxin-facilitated Amp dormants without observing a point of no return. Inactivation of *recA*, which suppresses the SOS response, marginally reduced the relative rate of antibiotic persistence. Transient ε expression reverses the negative effect of the toxin and indirectly awakes Amp dormants to levels observed in the *rec*^+^ control. This toxin-mediated sensitization of bacterial cells to Amp and the understanding of the conditions that inhibit antitoxin action will facilitate targeted engineering of their activity towards the development of anti-persistence agents.

## 2. Results and Discussion

### 2.1. Experimental Rationale

Resistance, tolerance, and persistence are independent solutions used by bacteria to survive an antibiotic action (Supplementary Figure S1) [1]. A bacterial population resistant to antimicrobial, which grows under drug pressure, uses mutation-associated defense mechanisms (such as antibiotic inactivation/modification, increased efflux, or target modification) to confer a resistant phenotype with or without a fitness cost (Figure S1) [19,44,45]. Persistence is a special case of tolerance, and often the two terms are interchangeable [45]. Tolerance is the general ability of a population to survive a longer antibiotic treatment without a significant change in the MIC (Figure S1, dotted line), whereas persistence represents a subpopulation of cells that can survive the antibiotic treatment (Figure S1, dotted vs. orange broken dotted line) [1].

The growth of susceptible bacteria is challenged by the addition of an antibiotic (e.g., Amp), but a subpopulation of stochastic variants, by undergoing a period of slow- or non-growth, escapes the antibiotic action, leading to a bimodal time-inactivation curve of cell persistence (Figure S1, orange broken dotted line) [1]. In *B. subtilis*, the mechanisms responsible for antibiotic persistence have not been delineated, and the fate of persistents after a prolonged exposure to the antibiotic before returning to permissive conditions is poorly understood. Here, Amp-induced cell-wall stress, by inducing *sasA* expression (one of the two small alarmone synthases), reduces the GTP pool and increases c-di-AMP and ppGpp levels [10,13,17]. This state might be transient and all cells might “awake” after removal of the antibiotic.

In previous studies, the number of toxin ζ and antitoxin ε molecules from a *S. pyogenes* plasmid-borne ε-ζ TA cassette was determined in the *B. subtilis* host grown in the minimal S7 medium [43,46]. An orthogonal control of ζ and ε was constructed by placing the ζ gene under the control of an isopropyl-β-D-thiogalactoside (IPTG) inducible promoter (*lacI*, *P_hsp_*ζ, *spc* cassette), was ectopically integrated into the *B. subtilis amy* locus (Table 1) [38]. The antitoxin ε gene, which reads from a xylose (Xyl)-inducible promoter (*xylR*, *P_xylA_*ε*, cat* cassette), was cloned into the middle-low-copy number pCB799 plasmid (~8 copies/cell) [38]. In *B. subtilis,* BG1125 pCB799 cells grown in S7 are prone to genome rearrangements, by transcriptional escape of the ζ gene from the *P_hsp_* promoter. The Xyl concentration required to reduce genome rearrangements was estimated to be 0.005% [10,38]. BG1125 (pCB799) grows in the S7 medium with 0.005% Xyl at 37 °C with a doubling time of (58 ± 2 min) (Figure S2) [38].

Then, the IPTG concentration necessary to express similar levels of toxin from the native system, and the Xyl concentration necessary to express sufficient ε_2_ to inactivate the toxin ζ action was estimated [38]. Transient toxin ζ expression, at or near physiological concentrations, induces reversible cell dormancy. Here, a large cell fraction enters into a toxin-induced dormant state that cannot form colonies since they minimize their metabolic activity. By contrast, a small subpopulation escapes the toxin ζ action without diminishing their metabolic activity. When the inducer of toxin expression was removed, the cells exited the transient dormant state and formed colonies, leading to bimodal toxin survivors (Figure S1, blue broken dotted line) rather than toxin tolerants (Figure S1) [15]. These cells that escape the toxin ζ action show no genetic changes [38].

Transient toxin ζ expression sensitizes cells to the Amp treatment [10,15]. In the BG1125 [pCB799] strain, the presence of IPTG or Xyl does not affect the MIC for Amp [10]. The fate of the Amp persisters, which are sensitized by toxin ζ is unknown. At least two types of mechanisms can be envisioned. First, toxin-induced dormancy sensitizes cells to Amp action. Second, ζ-induced dormancy and stochastic slow growth cells trigger a viable but not-culturable state. In the latter condition, normal culturable cells stochastically lost their ability to grow on media, but remain viable [47]. If the first hypothesis is correct, antitoxin ε expression inactivates ζ, and indirectly reverses Amp dormants, as depicted in Figure S1 (red dotted curve). However, if the latter hypothesis is correct, transient ε_2_ expression should reverse toxin-induced dormancy, but it should play no role on the exit from stochastic viable but the not-culturable state. These experiments performed here should contribute to understand the mechanism of genesis and exit of Amp persistence.

### 2.2. Toxin ζ Induces a Slow-Growth Active State

In γ-Proteobacteria, deletion of chromosomally-encoded type II TA loci neither confers a fitness benefit nor influences stress tolerance [48,49]. Similarly, deletion of *B. subtilis*-encoded type II TA systems has no effect on antibiotic persistence [12]. To gain insight on the molecular and physiological bases of toxin-induced dormancy, dormant cells were infected with a lytic bacteriophage (phage) and the infective cycle was followed.

Except TA systems that abort infection of a specific phage in selected hosts [50], and those phages that encode *bona fide* TA systems [23], it has been observed that overexpression of type II toxins contributes to abortive infection and causes exclusion of lytic phages from the bacterial population [26,51]. It has been also shown that in the arms race of a bacterium and its phage, a λ lytic variant was able to infect and kill survival bacteria, despite their dormancy state [52], and that T4 infection inactivates MazF toxin activity [53]. In contrast, it has been also shown that phage λ cannot overcome a toxin treat, since toxin overexpression, acting as a defense mechanism, protects the bacterial culture by increasing (p)ppGpp levels, and the alarmone might antagonize λ phage development [54]. Moreover, toxin RnlA overexpression cleaves phage T4 mRNAs to antagonize T4 amplification [55]. Therefore, phage infection of toxin-induced dormant cells can be a powerful tool to understand the physiology of toxin-induced dormancy and to address how the toxin ζ expression sensitizes cells to toxin ζ expression.

**Figure 1:**
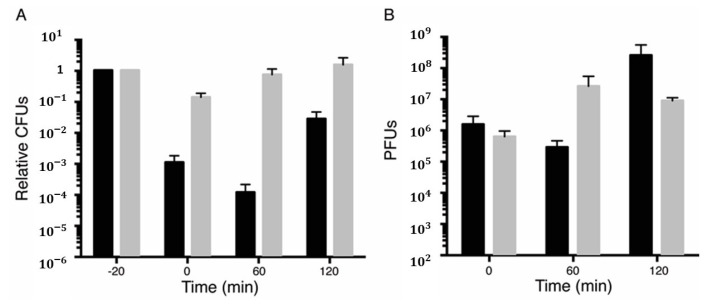
Toxin dormants infected with the lytic phage SPP1 are metabolically active. Survival rate of BG1125 (pCB799) cells infected by the SPP1 phage after ζ toxin induction. BG1125 (pCB799) cells were grown in the minimal S7 medium, containing traces of Xyl (0.005%) and 10 mM MgCl_2_ to OD_560_ = 0.2 at 37 °C. At this time (-20 min) the culture was divided in two. To one half, IPTG (2 mM) was added (black bars) to induce ζ expression (-20 min) and incubated for 15 min. After 15 min, phage SPP1 at *moi* = 1 was added to both cultures and cells were incubated for further 5 min. At 0 min, cells were centrifuged to remove unadsorbed phages and resuspended in S7 medium, containing 0.005% Xyl and 10 mM MgCl_2_. Colony-forming units (CFUs) were counted plating on a LB agar (time 0) and both infected cultures were incubated for further 60 min (60 min). At 60 min, 0.5% Xyl was added to induce antitoxin ε expression to neutralize toxin action, and both infected cultures were incubated for further 60 min (120 min). At 0, 60, and 120 min, samples were withdrawn and plated in LB agar plates lacking IPTG to count CFUs (**A**), or in a lawn of exponentially growing BG214 cells onto LB plates supplemented with 10 mM MgCl_2_ to measure PFUs (**B**). Data are shown as mean ± standard error of the mean (SEM), from >3 independent experiments.

**Figure 2:**
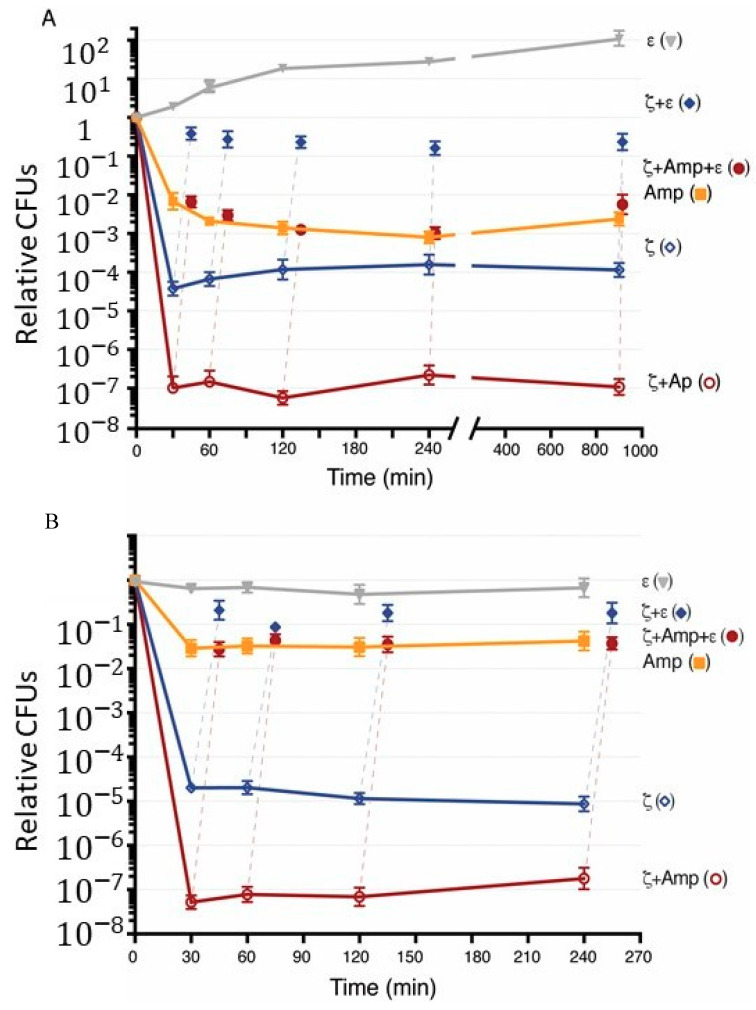
Toxin ζ induces reversible dormancy, and facilitates Amp dormancy. (**A**), BG1125 (pCB799) cells were grown in the S7 medium, containing traces of Xyl (0.005%) at 37 °C and the cultures were divided. Then, IPTG (2 mM) to induce ζ expression, Amp (at 2× MIC, 3 μg mL^−1^), or both IPTG and Amp were added (0 min), and the cultures were incubated for 900 min. At various times, samples were withdrawn, centrifuged, and plated on LB agar plates to count ζ (empty blue rhomb), Amp (filled orange square), or both ζ and Amp survivals (empty purple circles). At 30, 60, 120, 240, and 900 min, aliquots were taken and 0.5% Xyl was added to induce antitoxin ε expression, and the cultures were incubated for 15 min before plating in LB agar plates containing Xyl, but lacking IPTG (filled blue rhomb) or both IPTG and Amp (filled purple circle). The vertical broken lines join the original point (ζ or ζ + Amp) with the reversed condition after ε expression. (**B**) An overnight culture of BG1125 (pCB799) cells grown in the S7 medium, containing traces of Xyl (0.005%) was normalized to ~1 × 10^9^ cells mL^−1^ (37 °C) and the cultures were divided. Xyl (0.5%) to induce ε expression as the control was added. IPTG to induce toxin, Amp, or both IPTG and Amp was added (0 min), and the cultures were incubated for 240 min. At various times, samples were withdrawn and plated in LB agar plates lacking IPTG (empty blue rhomb), Amp (filled orange square), or both IPTG and Amp (empty purple circles). At various times, 0.5% Xyl was added to induce antitoxin expression, and the cultures were incubated for 15 min before plating. Samples were withdrawn and plated in LB agar plates containing Xyl, but lacking IPTG (filled blue rhomb) or both IPTG and Amp (filled purple circle). The vertical broken lines join the original point (ζ or ζ + Amp) with the reversed condition after ε expression. Data are shown as mean ± standard error of the mean (SEM), from >4 independent experiments.

A set of isogenic *B. subtilis* strains, lacking prophages (SPβ and PBSX), conjugative genetic elements, CRISPR-Cas and restriction systems, and having a deficiency in the *rsbV* gene, whose function is crucial for the activation of RNA polymerase σ^B^-mediated general stress response [56], were used to characterize the impact of toxin ζ expression on cell dormancy (Table 1). The BG1127 (pCB799) strain (in the presence or absence of IPTG, doubling time 59 ± 3 min) and BG1125 (pCB799) strain (in the absence of IPTG, doubling time 58 ± 2 min) grown in the minimal S7 medium supplemented with traces of Xyl (0.005%) at 37 °C with shaking had a similar doubling time (Figure S2). Both cell cultures reached a plateau at ~3.5 × 10^9^ cells mL^−1^ (Figure S2). By contrast, if IPTG (2 mM) was added at OD_560_ = 0.2, the growth curve of the BG1125 (pCB799) strain was halted and after 720 min the OD_560_ was not significantly decreased (Figure 1 and Figure S2), but the plating efficiency was significantly reduced (Figure 2A) [38].

BG1125 (pCB799) cells were grown in the minimal S7 medium supplemented with 0.005% Xyl, to OD_560_ = 0.2 at 37 °C with shaking. At this time (-20 min), the culture was divided, 2 mM IPTG (time zero) was added to a half, and both cultures were incubated for 20 min to allow toxin-induced dormancy. The total number of cells were measured as colony-forming units (CFUs) by plating them on LB plates lacking IPTG (Figure 1A and Figure S3). At -5 min, both cell cultures, containing or not IPTG, were infected with phage SPP1 at a multiplicity of infection (*moi*) of 1 (to minimize the noise of free phages), and incubated for further 5 min. Then, free phages were removed by centrifugation, and this time was considered as 0 min in our experiment. At 0 min, the number of cells was measured by plating on LB agar plates and plaque-forming units (PFUs), to determine the number of infected centers, cells were measured by plating appropriate dilutions of the culture in a lawn of exponentially growing BG214 cells (indicator strain) onto LB-Mg (supplemented with 10 mM MgCl_2_) plates (Figure 1A,B). In parallel, free phages from the supernatant of centrifugation were determined, to test whether centrifugation was sufficient to remove them or polyclonal anti-SPP1 antibodies were necessary to the inactive free phages. Since in the minus IPTG condition the number of total cells minus free phages was not significantly different from the number of infected centers, we have omitted the use of polyclonal anti-SPP1 antibodies.

After 20 min of toxin induction IPTG, CFUs were reduced by ~1000-fold at a time when compared to the parallel culture in the absence of IPTG (Figure 1A and Figure S3, black vs. grey bar). After infection with SPP1, the proportion of infected centers measured as PFUs was similar in the minus and plus IPTG conditions (Figure 1B, black and grey bars), suggesting that a large fraction of dormant cells were infected by SPP1. In the absence (grey bar) and presence of IPTG (black bar), the phage titer, which correlates to the cell population infected by SPP1, was similar and in good agreement with the expected for a one-hit kinetic. From the input phages, ~71% and ~67% infected the cells, respectively, defining the infected centers (Figure 1B) and ~30% were recovered from the supernatant after centrifugation. In parallel, the total number of cells at -20, 0, 60, and 120 min in the absence of phage SPP1 was also measured as CFUs by plating them on LB plates lacking IPTG (Figure S3).

The SPP1 infection cycle takes 35–45 min in cells growing in the minimal medium, thus the infected cells were incubated for 60 min, and then the relative number of progeny phage per infected cell (burst size) was deduced for the minus and plus IPTG conditions. At 60 min and in the presence of IPTG, cells remained in the dormant state (Figure 1A vs. Figure S3), suggesting that SPP1 does not encode (a) gene(s) whose function can neutralize toxin ζ action. At 60 min, the phage SPP1 titer from cultures where the toxin had been induced was similar to the number of infected cells at 0 min. However, in the absence of IPTG, the PFUs greatly increased. The SPP1 relative burst size (the ratio of the total number of SPP1 progeny to the number of input phage) from three independent experiments was quantified by counting cells and phages taking in to account the supernatant of the cultures (Figure 1A,B). The SPP1 burst size significantly increased (~80 SPP1 phages/infected cell). These results suggest that SPP1, upon infecting toxin-dormant cells neither enters in the amplification cycle nor promotes the exit of the dormant state.

To test whether dormant cells antagonize or cannot support SPP1 amplification or if dormant cells can support SPP1 amplification upon toxin neutralization at 60 min post-infection, Xyl (0.5%) was added to induce antitoxin ε expression, and the cultures were incubated for further 60 min. At 120 min, the number of total cells and the SPP1 burst-size were quantified as described above. In the presence of IPTG and Xyl, the yield of SPP1 phage in cells expressing both ζ and ε genes (between 60 to 120 min) was significantly increased (~110 SPP1 phages/predicted infected cell), calculated from the PFU obtained and the number of infected centers at 60 min (Figure 1B). This suggests that only when infected cells exit toxin-induced dormancy, by the anti-toxin action, SPP1 was amplified. In the only Xyl condition (i.e., no toxin induced), the number of total cells increased (58 ± 2 min doubling time), but the phage titer did not, suggesting that these cells enter in a state that might not be further infected by the SPP1 phage.

These data altogether suggest that: (i) SPP1 infected toxin-induced dormant cells with a similar efficiency as normally growing cells (Figure 1B, time zero); (ii) ζ-induced dormancy does not impair phage absorption, but delays the SPP1 lytic cycle; (iii) toxin ζ-induced dormancy is a metabolically active state rather than a growth-arrested state leading to abortive infection; (iv) SPP1 does not undergo an abortive infection; and v) upon toxin ζ inactivation, by antitoxin ε expression, the exit of ζ-induced dormancy facilitates a viral infective cycle.

### 2.3. Discrete Subpopulations of Toxin ζ Survivors and Amp Persisters

The subpopulation of cells that survive an antibiotic treatment, are termed persisters [1,45]. The rate of killing by Amp is strictly proportional to the rate of bacterial growth, and Amp persistence is associated with a pre-existing phenotypic switching to a stochastic non-growing state (or leftover cells from high-stress stationary-phase inoculums) [57,58]. To analyze the interconnection between toxin survivors and Amp persisters and whether a prolonged growth arrest causes an irreversible cell cycle arrest (point of no return), we have used BG1125 (pCB799) cells (Table 1).

BG1125 (pCB799) cells were grown in the minimal S7 medium, supplemented with 0.005% Xyl, to OD_560_ = 0.2 at 37 °C with shaking, and then 2 mM IPTG (0 min) was added to induce toxin expression, and the reaction incubated for 900 min (Figure 2A). Toxin ζ expression quickly reduced the cell plating efficiency showing a biphasic time-inactivation curve (Figure 2A, blue empty rhombs), suggesting that toxin ζ expression reaches a steady-state in a short time and it remains for at least 900 min. It is likely that toxin ζ expression induces a slow-growth active state, but a rare fraction (3–6 × 10^−5^) exits this dormant state and survives, or a preexisting population of non-growing cells are insensitive to toxin action (type I-like persisters) (see [59]). Alternatively, expression of the ζ gene triggers the expression of any of the type I and/or type II TA modules present in the background, and the loss of cell viability and the observed surviving cells arise via the overlapping activation of multiple toxins, even with different activities.

To test whether the frequency of survivors may increase in response to the spontaneous induction of other chromosomally encoded toxins present in the background, IPTG was removed, by washing the culture with the pre-warmed S7 medium, containing 0.5% Xyl (to induce antitoxin ε expression), and cells were preincubated for 15 min before plating on LB plates containing 0.5% Xyl, but lacking IPTG. Antitoxin ε expression reversed ζ-induced dormancy, by significantly increasing cell viability nearly to full values almost similar to the time of IPTG addition (Figure 2A, blue filled rhombs). Similar results are observed when IPTG is not removed from the medium [15]. It is likely that: (i) Toxin ζ-induced dormancy rather than cell lysis, as judged by the non-significant variation in the OD_560_ = 0.2 during a 720-min interval (Figure S2); (ii) toxin ζ is not triggering a spontaneous induction of toxins from TA systems present in the background; and (iii) toxin ζ transiently halts DNA replication and triggers a phenotypic heterogeneity [38]. In addition, the expression of the antitoxin ε expression reversed ζ-induced growth arrest even after a prolonged dormancy (equivalent to ~15 mass doubling periods) (Figure 2A, blue filled rhombs).

To address whether antibiotic persisters are produced due to a stochastic entrance into the stationary phase, the overnight culture was extensively diluted and BG1125 (pCB799) cells were grown in the minimal S7 medium, supplemented with 0.005% Xyl, to OD_560_ = 0.2 at 37 °C with shaking. It is expected that <0.3% of the total cells in the actively growing inoculum are old cells from the stationary-phase inoculum. Amp (at 2× MIC, 3 μg mL^−1^) was added, and the culture was maintained up to 900 min. At indicated times, the cells were washed to remove the antibiotic, and plated in LB lacking Amp. The removal of Amp was sufficient to reveal that a large fraction of cells (>99% of total cells) cannot form colonies upon exposure to bacteriolytic Amp (Figure 2A, filled orange squares). As stated, the rate of Amp killing is proportional to the rate of bacterial growth [57], with >80% of total Amp-treated cells being stained with propidium iodide [10], which stains the membrane compromised cells. A small cell fraction (1–0.5 × 10^−2^ survivals), however, regained the ability to form colonies upon antibiotic removal (Figure 2A, filled orange squares). Similar results were observed when the overnight culture was less extensively diluted (~6% old cells), suggesting that old cells from the stationary-phase inoculum poorly contribute to the Amp persistence fraction.

Upon exposure to Amp for at least 900 min of incubation, cells survive following antibiotic removal (Figure 2A) rather than becoming metabolically inactive by entering a path of no return as described upon toxin MazF overexpression [60]. The culturable cells were still sensitive to Amp action, thus we have to believe that after prolonged incubation to the antibiotic there were no genetic changes [10].

### 2.4. Transient Toxin ζ Expression and Amp Addition Reduce the Subpopulation of Persisters

Transient toxin ζ expression sensitizes cells to the presence of different antibiotics [10,37]. At least two mechanisms can be envisioned: (i) Toxin dormants are sensitive to antibiotic action and stochastically enter in a viable but not-culturable state with resuscitation independent of antitoxin expression; or (ii) antibiotic persistence is the sum of stochastic and transient antibiotic-induced dormants with resuscitation dependent of antitoxin expression. To test the above hypotheses, BG1125 (pCB799) cells were grown in S7 medium, supplemented with 0.005% Xyl, to OD_560_ = 0.2 at 37 °C with shaking. Then, 2 mM IPTG and 3 μg mL^−1^ Amp were added, and at indicated times cells were washed and plated in the absence of both IPTG and Amp. A typical biphasic decline curve was observed (Figure 2A, purple empty circles). Amp and toxin action significantly decreased the proportion of survivals (1–2 × 10^−7^) when compared to only ζ (3–6 × 10^−5^ survivors) or only Amp (1–0.5 × 10^−2^ persisters) (Figure 2A, purple empty circles vs. blue empty rhombs and orange filled squares). The persistent cells stochastically switch back to a growing state, upon plating in the absence of IPTG and Amp, with the fraction of persisters remaining nearly constant for ~900 min (Figure 2A, purple empty circles). It is likely that: (i) Amp persisters are in an active state that allows toxin expression; (ii) toxin ζ expression does not increase antibiotic persistence; (iii) toxin expression triggers a network of intracellular stress responses that significantly increases cell dormancy; and (iv) toxin-induced dormancy facilitates Amp dormants that resuscitate upon antitoxin expression.

### 2.5. Transient Antitoxin ε Expression Reverses ζ-Facilitated Exit of Amp Dormants

To test whether transient ζ expression enhances Amp efficacy with the cells entering in a viable but not-culturable state or if ζ expression induces dormancy of Amp persisters, BG1125 (pCB799) cells were grown in the minimal S7 medium, supplemented with 0.005% Xyl, to OD_560_ = 0.2 at 37 °C with shaking. Then, 2 mM IPTG and Amp (3 μg mL^−1^) were added and the cells incubated up to 900 min (Figure 2A, purple empty circles). At indicated times, IPTG and Amp were removed by washing the culture with the pre-warmed S7 medium, containing 0.5% Xyl to induce antitoxin ε expression, and cells were preincubated (15 min) before plating (Figure 2A, purple filled circles). The antitoxin ε recovered cell proliferation to levels equivalent to that of Amp persisters, even after 900 min of toxin ζ and Amp action (Figure 2A, purple filled circles). These data altogether suggest that: (i) Toxin ζ expression and Amp addition induce prolonged dormancy (at least for 15 h) rather than programmed-cell death, as reported for the *E. coli* MazEF and RelBE locus (reviewed in [61]); (ii) ζ-induced dormancy is insensitive to Amp action; (iii) toxin-induced dormant cells awake and form colonies even after 15 h of toxin and Amp action, rather than triggering cell lysis or inducing a viable but not-culturable state (Figure 2A, purple filled circles); and (iv) the orthogonal control of ζ and ε_2_ levels revealed that a TA system can be an important element in coping with Amp stress; and (v) toxin survivors and Amp persistence are not epistatic.

### 2.6. Toxin ζ Expression Induces Amp Dormancy in Non-Growing Cells

To re-evaluate whether Amp persisters are produced due to a stochastic entrance into the stationary phase or upon transient toxin ζ expression, and if toxin expression or Amp addition to stationary phase cells affects the persistence level and its subsequent exit, high-density slow- or non-growing stationary phase *B. subtilis* BG1125 (pCB799) cells, which grew overnight in the S7 medium supplemented with 0.005% Xyl, were used. The overnight culture was normalized to ~1 × 10^9^ cells mL^−1^ with the pre-warmed S7 medium, supplemented with 0.005% Xyl and split. Amp (3 μg mL^−1^), IPTG (2 mM), or both Amp and IPTG were added and the cells incubated up to 240 min. At the indicated times, IPTG, Amp, or both were removed from the medium and the culture was plated on LB plates. Since in the absence of both, Amp and IPTG, the number of cells did not significantly increase at 240 min (Figure 2B, filled grey triangles) and BG1125 (pCB799) cells grown in S7 supplemented with 0.005% Xyl usually had an usual lag-time of 30–45 min (Figure S2), we assume that the high-density cells remain in the stationary phase during the experimental period.

The MIC for Amp was estimated using low-density cells (1–3 × 10^6^ cells mL^−1^ for 16 h, at 37 °C), and no correction by per-cell in the Amp concentration was performed. At a variable time, and upon removal of the antibiotic, a significant fraction could not form colonies, but a subpopulation of cells persisted to Amp action (3–5 × 10^−2^ survivals). As expected, the proportion of persisters was significantly higher than in exponentially growing cells (Figure 2A vs. Figure 2B, filled orange squares). This is consistent with the observation that the rate of killing of Amp is strictly proportional to the rate of bacterial growth [57]. Similar results were observed in the presence of 6 μg mL^−1^ Amp (data not shown). It is likely that the lower proportion of cell death might correlate with the less proportion of metabolically active cells in the stationary phase, but in the only Xyl condition the cells are not proliferating.

In the presence of IPTG, the subpopulation of toxin ζ (3–6 × 10^−5^ survivals) did not significantly increase during time and was similar in both, exponential growth and stationary phase (Figure 2A,B, empty blue rhombs). Similar results were observed when a low-lived ζ variant (ζY83C) is used [10], suggesting that when toxin ζ is expressed, it triggers dormancy in high-density stationary phase cells. When Amp and IPTG were added, a low proportion of high-density stationary phase cells survive toxin ζ and Amp action (4–7 × 10^−8^ survivals) (Figure 2B, empty purple circles).

To test whether the expression of ε antitoxin also reverses the effect of ζ-mediated dormancy in high-density non-growing cells, Xyl was added at the indicated times. The expression of ε antitoxin reversed the effect of the ζ toxin (3–5 × 10^−1^ survivals) and partially reversed toxin and Amp (4–5 × 10^−2^ survivals) (Figure 2B, filled blue rhombs and purple circles). It is likely that: (i) Toxin ζ induces a reversible dormant state in high-density, non-growing cells rather than entering in a stochastic viable but not-culturable state; (ii) there are two different subpopulations of Amp persisters: Stochastic (3–5 × 10^−2^ survivals) and toxin-induced (4–7 × 10^−8^ survivals); and iii) antitoxin expression reverses the apparent sensitization of toxin dormants to Amp action.

### 2.7. RecA Inactivation Does not Alter the Antitoxin Awake of Toxin and Amp Dormants

Toxin-induced dormants are metabolically active slow-growing cells, and this may enhance the development of heritable genetic changes, since many of the stress-response programs involved in the survival of persisters can also accelerate genome-wide mutagenesis [58]. In Proteobacteria, both stochastic and stress-induced slow-growth persisters have been shown to depend on the SOS responses [58,62]. Furthermore, the SOS response induces the expression of certain type I and II TA modules in a subpopulation of *E. coli* [5,63].

In Proteobacteria and Firmicutes, bactericidal antibiotics (e.g., Amp), regardless of the drug-target interaction, induce changes in cellular metabolism that promote the production of highly deleterious hydroxyl radicals, leading to cell killing [64]. Amp-induced hydroxyl radicals, may damage template bases and introduce single strand nicks. These nicks, through DNA replication, can be converted into one-ended double-strand breaks (DSBs), that cause a significant induction of the SOS response [64,65] and maintenance of Amp persistence in *E. coli* cells [66]. However, DNA damage caused by sublethal doses of a fluoroquinolone in *E. coli* promotes persistence, but not Amp [5]. The recombinase *recA*, which is a central player in the SOS response, contributes to the repair of the damaged template bases that escape specialized repair, and of the nicks that are converted in one-ended DSBs during DNA replication [67,68]. The contribution of the SOS response to the DSB repair, however, differs substantially between *E. coli* and *B. subtilis* cells. In *B. subtilis*, the inability to induce the SOS response does not compromise the DSB repair [69].

In *E. coli* cells, *recA* plays a critical role in the appearance of apoptotic phenotypes and in pushing the cell towards its death [65], and inactivation of *recA* potentiates killing by different antibiotics, Amp among them [64]. In *B. subtilis* cells, transient toxin ζ expression [38,43] or extended cell cycle arrest [70] does not induce the SOS response. To test whether *recA* inactivation sensitizes cells to toxin or Amp, a Δ*recA* mutation was mobilized onto the *B. subtilis* BG1125 (pCB1226) strain by SPP1-mediated generalized transduction, rendering the BG1889 (pCB1226) strain (Table 1). Plasmid pCB1226 is a derivative of pCB799, but the *erm*C gene was selectively inactivated so that the Δ*recA* mutation could be introduced in *B. subtilis* BG1125 (pCB1226) cells by Erm^R^ selection (Table 1).

**Figure 3:**
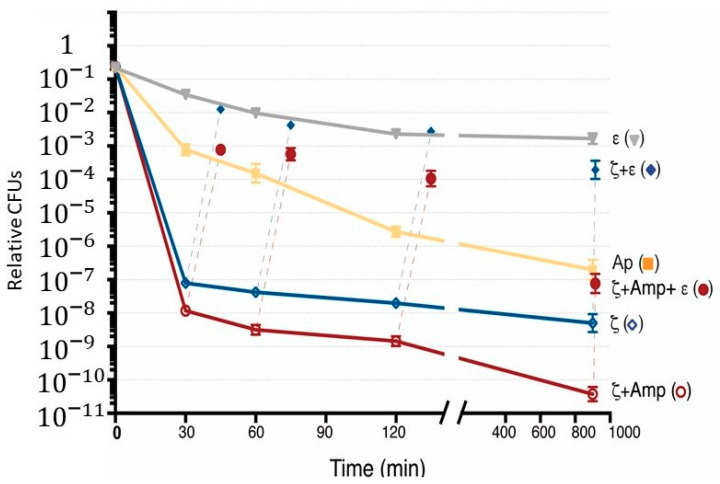
Inactivation of *recA* does not affect the frequency of ζ survivors and Amp persisters. BG1889 (pCB799) cells were grown in the minimal S7 medium, containing traces of xylose (Xyl; 0.005%) to OD_560_ = 0.2 (with CFUs of 1–3 × 10^6^ cells mL^−1^) at 37 °C. Then, the cultures were divided. To the indicated cultures, Xyl (0.5%) to induce ε expression as the control, IPTG (2 mM) to induce ζ expression, Amp (3 μg mL^−1^) or both IPTG and Amp were added (0 min), and the cultures were incubated for 900 min. At various times, samples were withdrawn and plated in LB agar plates. At various times, aliquots were taken and 0.5% Xyl was added to induce antitoxin expression, and the cultures were incubated for 15 min before being plated in LB agar plates containing Xyl, but lacking IPTG (filled blue rhomb), or both IPTG and Amp (filled purple circle). Data are shown as mean ± standard error of the mean (SEM), from >4 independent experiments.

BG1889 (pCB1226) cells were grown in S7 medium, supplemented with 0.005% Xyl, to OD_560_ = 0.2 at 37 °C with shaking. CFUs at this OD_560_ are 3–6 × 10^6^ cells mL^−1^, in agreement with previous reports showing that in *B. subtilis* only 10–20% of Δ*recA* cells can form colonies in plates in the absence of DNA damage [71]. Then, IPTG (2 mM), Amp (3 μg mL^−1^), or both IPTG and Amp were added (0 min), and the culture was maintained for 900 min (Figure 3). In the absence of ITPG and presence of traces of Xyl (0.005%), the BG1889 (pCB1226) cells had a poor fitness, formed small-size colonies, and the plating efficiency was reduced ~100-fold after 900 min of incubation (Figure 3, filled grey triangles). This result indicates that a significant proportion of Δ*recA* cells may have a prolonged defect in DNA repair and chromosomal segregation, which reduces cell viability during prolonged growth.

The number of Amp persisters and toxin survivors during the first 120 min was lower ~2 × 10^−3^ and ~4 × 10^−6^, respectively, but persistence to both Amp and toxin (~4 × 10^−7^ frequency of persisters) in the Δ*recA* context (Figure 3, orange filled squares and blue empty rhomb vs. grey filled triangles) was similar to that of the *rec*^+^ control (see Figure 2A). The decrease of toxin survivors and Amp persisters was observed at 900 min (~7 × 10^−9^) after correction of the poor viability of the BG1889 (pCB1226) strain in the absence of IPTG and Amp (Figure 3, purple empty circles vs. grey filled triangles). What is the significance of such large variations in persisters to both toxin and Amp action in the Δ*recA* context? First, to measure persisters, the culture was concentrated ~100-fold. Second, under this experimental condition, the relative persistence frequency is similar to the spontaneous mutation rate. Finally, *B. subtilis* has multiple forms of differentiation and development, thus we cannot rule out that any uncontrolled condition might affect the outcome.

Transient antitoxin ε expression, by the addition of Xyl, reversed toxin action and the plating efficiency was increased to levels similar to the control strain in the absence of any treatment (Figure 3, blue filled rhombs vs. grey filled triangles). When Xyl was added to cells treated with both Amp and IPTG, the expression of ε antitoxin reversed the effect of ζ toxin and facilitated the exit of Amp dormants (Figure 3, purple filled circles). It is likely, therefore, that: (i) *RecA* inactivation, which impairs the SOS response, does not significantly increase the number of Amp or toxin survivors; (ii) *recA* inactivation reduces toxin or Amp persistence, suggesting that Amp-induced hydroxyl radicals accumulation is not significantly deleterious when compared to the *recA* control without Amp and IPTG (Figure 3, orange filled squares and blue empty rhombs vs. grey filled triangles); and (iii) *recA* does not induce an apoptotic phenotype upon Amp treatment, and *recA* inactivation does not sensitize cells to both toxin ζ and Amp action.

## 3. Conclusions

This study provides new insights that allow us to unravel the mechanisms underlying the mode of action of the toxin ζ dormancy and indirectly of toxin and Amp dormants. Transient toxin ζ expression, at or near physiological concentrations, induces a slow-growth dormant state, and a small fraction of cells (persisters) is insensitive to the toxin action in *rec*^+^ and *recA* backgrounds. Toxin ζ-induced dormant cells can be infected with the lytic phage SPP1 with a similar efficiency as exponentially growing cells, but the amplification cycle is halted. Neutralization of the toxin, by inducing antitoxin expression, allows dormant cells to exit this state and this allows phage amplification. It is likely that toxin dormants are metabolically active rather than in a growth-arrested state.

Amp action kills ~99% of susceptible cells, but stochastic Amp persisters (frequency of 1–0.5 × 10^−2^) can form colonies upon removal of the antibiotic. Toxin survivors are significantly rare (~3 × 10^−5^), and in the presence of Amp the proportion of bacterial Amp and toxin survivors was further reduced (by 100–200-fold), suggesting a non-epistatic effect. To further evaluate the viable but non-culturable state hypothesis, we inactivated *recA*. In *E. coli* cells, *recA* contributes to apoptotic cell death in Amp treated cells [64], but the proportion of Amp persisters was not affected in the absence of *recA* [5]. In *B. subtilis* cells, toxin ζ and Amp persistence are not significantly affected in *recA* cells when compared to *rec*^+^ control, and transient antitoxin ε expression is necessary and sufficient to switch off toxin-induced dormancy, and to awake the fraction of toxin-facilitated Amp-induced persisters both in the *rec*^+^ and *recA* cells. We propose that in exponentially growing cells, two different subpopulations of Amp persisters are found, stochastic and toxin-induced dormancy, that also induce long term Amp dormancy.

## 4. Materials and Methods

### 4.1. Bacterial Strains and Plasmids

The bacterial strains and plasmids used in this study are listed in Table 1. All *B. subtilis* strains are isogenic with BG214. The strain BG1125 bearing *lacI*-*P*_hsp_ *wt*ζ and pCB799-borne *xylR*-*P*_xylA_ *wt*ε *ermC cat* cassette were previously reported (Table 1) [38]. The BG1885 strain was constructed in three steps. First, by site-directed mutagenesis (QuickChange Kit, Stratagene) using pCB799 as a template, the *ermC* gene was inactivated to render pCB1226. Second, the *lacI*-*P*_hsp_ *wt*ζ cassette was mobilized into BG214 cells bearing pCB1226-borne *xylR*-*P*_xylA_ *wt*ε *cat* cassette with selection for Cm^R^. Third, the null *recA*::*Erm* gene was mobilized by chromosomal transformation into competent BG214 (pCB1226) cells with selection for Erm^R^ (Table 1). Integration by double crossover was analyzed by PCR.

Toxin ζ gene expression (transcribed from *P*_hsp_) is regulated by IPTG (Calbiochem, Madrid, Spain) addition and the expression of ε gene (transcribed from *P*_xylA_) is regulated by Xyl (Sigma-Aldrich, St. Louis, MO, USA) addition [38].

### 4.2. Growth Conditions

BG1125 (pCB799) and BG1125 (pCB1226) cells were grown to a mid-exponential phase (OD_560_ = 0.2) in S7 medium supplemented with methionine, tryptophan, and 0.005% Xyl at 37 °C with shaking [43]. Under this condition, cells grew in S7 medium, with a doubling time of 50–60 min. Transient toxin and/or antitoxin expression was induced by IPTG (2 mM) and/or Xyl (0.5%) addition. Before plating, cells were centrifuged and resuspended in the fresh LB medium to remove the inductor or the antibiotic, and dilutions were plated on LB agar plates. The survival rate was derived from the number of CFUs in a given condition relative to the CFU of the non-induced/non-antibiotic-treated control. All plates were incubated for 20 h at 37 °C.

The lytic SPP1 phage was used to test the toxin effect. BG1125 bearing *lacI*-*P*_hsp_ *wt*ζ and pCB799-borne *xylR*-*P*_xylA_ *wt*ε gene were grown to a mid-exponential phase at 37 °C in S7 medium, supplemented with methionine, tryptophan, 0.005% Xyl, and 10 mM MgCl_2_. Toxin expression was induced by adding IPTG 2mM and after 15 min cells were infected with phage SPP1 at a multiplicity of infection (*moi*) of 1 in the presence or absence of IPTG. After 5 min, cells were centrifuged to remove unabsorbed phages and resuspended in S7 medium with 10 mM MgCl2 (0 min) and the culture was incubated for 60 or 120 min. The inducers were removed by washing, and the phage titer (PFUs) was calculated by plating the appropriate dilution deposited in a lawn of exponentially growing BG214 cells onto LB plates supplemented with 10 mM MgCl_2_ (LB-Mg) to measure PFUs as described [72]. The plates were then incubated for 18–20 h at 37 °C.

The MIC for Amp (Sigma-Aldrich, St. Louis, MO, USA) was estimated by exposing 1–3 × 10^6^ cells mL^−1^ (16 h, 37 °C) in the minimal S7 medium, with an increasing Amp concentration under shaking (240 rpm). The minimal concentration that gave no growth overnight (1.5 μg mL^−1^) was defined as MIC. The Amp concentration used was twice the MIC (2× MIC) or 3 μg mL^−1^.

## Figures and Tables

**Table 1:** Bacterial strains used.

Strain	Relevant Genotype	Reference
BG214	*trpCE metA*5 *amyE1 ytsJ*1 *rsbV*37 *xre*1 *xkd*A1 *att*^SP^^β^, *att*^ICE*Bs*1^	Lab. Collection
BG1127	+ *lacI*, *P_hsp_* ^a^, *spc,* [pCB799-borne *xylR, P_xylA_*ε ^b^, *ermC*, *cat*]	[38]
BG1125	+ *lacI*, *P_hsp_*ζ ^a^, *spc,* [pCB799-borne *xylR, P_xylA_*ε ^b^, *ermC*, *cat*]	[38]
BG1125	+ *lacI*, *P_hsp_*ζ ^a^, *spc,* [pCB1226-borne *xylR, P_xylA_*ε ^b^, *cat*]	This work
BG1889	+ *lacI*, *P_hsp_*ζ ^a^, *spc,* Δ*recA* (*recA*:*erm*) [pCB1226-borne *xylR, P_xylA_*ε ^b^, *cat*]	This work

All *B. subtilis* strains are isogenic with the BG214 strain. ^a^ The *P_hsp_* promoter is under the control of the LacI repressor. ^b^ The *P_xylA_* promoter is under the control of the XylR repressor.

## Supplementary Materials

The following data are available online at https://www.mdpi.com/2072-6651/12/12/801/s1, Figure S1: Graphic illustration of the distinct responses that may occur after Amp and toxin ζ action, Figure S2. Growth curves of BG1127 (pCB799) and BG1125 (pCB799) strains in the presence or absence of IPTG, Figure S3. BG1125 (pCB799) in the presence or the absence of IPTG.
