# Supplementary material for: Antitoxin ε Reverses Toxin ζ-Facilitated Ampicillin Dormants
Source: Toxins (Basel). 2020 Dec 15;12(12):801. doi: 10.3390/toxins12120801 (PMC7765365; doi:10.3390/toxins12120801)
Supplement: Supplementary file 1 [file toxins-12-00801-s001.pdf]

Supplementary Materials: Antitoxin ε₂ reverses toxin ζ-facilitated ampicillin dormants

María Moreno-del Álamo, Chiara Marchisone and Juan C. Alonso

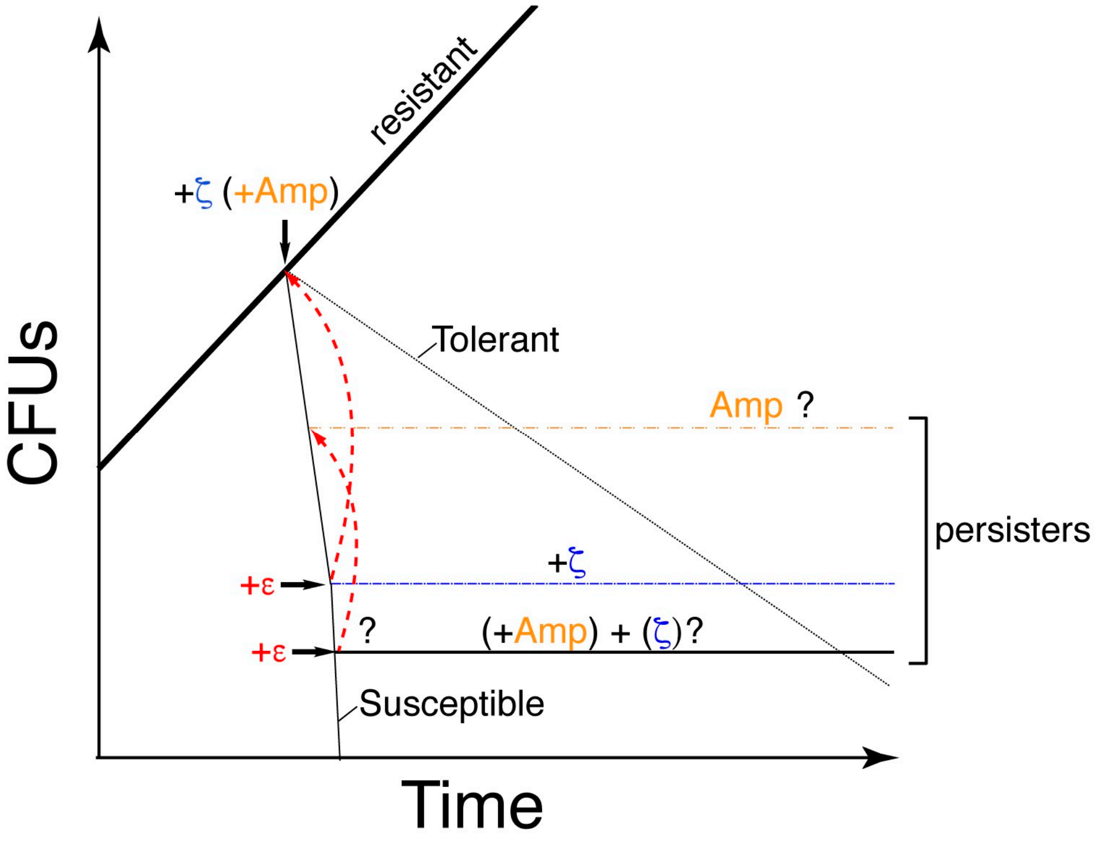

Figure S1. Graphic illustration of the distinct responses that may occur after Amp and toxin z action. Proliferation of a resistant clone is not affected (thick black line), but growth of the susceptible clonal cell is halted by transient toxin z expression (upon IPTG addition, [+z, blue]) or Amp addition at 2xMIC (+Amp, orange broken dotted line). Potential outcomes after IPTG or Amp addition: (i) a large fraction of cells is susceptible to the drug (black line); (ii) a subpopulation persists/survivors and forms colonies, leading to a biphasic time-inactivation curve ([+z, blue] or [(+Amp, orange]); (iii) transient expression of antitoxin e_2 (+e_2, red) awakes the cells susceptible to toxin z action (dotted red line); (iv) transient toxin z expression and Amp addition yield distinct persists/survivors subpopulations with an unknown outcome (red broken line); and (v) a fraction of tolerants cells showing a linear time-inactivation curve (dotted line) was not observed.

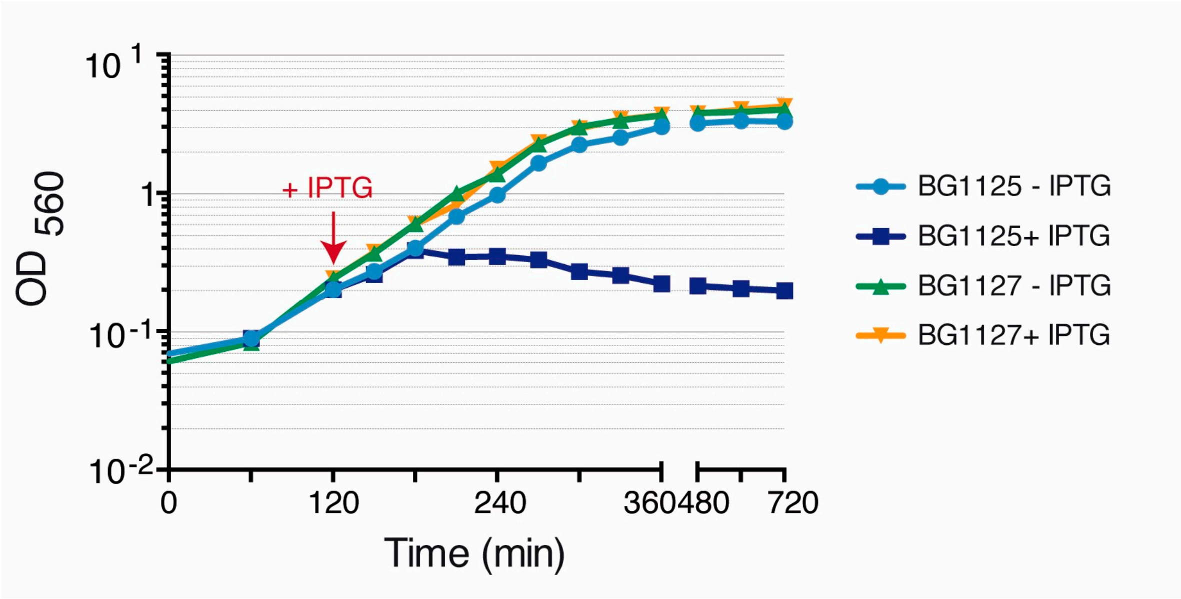

Figure S2. Growth curves of BG1127 (pCB799) and BG1125 (pCB799) strains in the presence or absence of IPTG. Overnight cultures grown in S7 medium, containing traces of Xyl (0.005%) at 37 °C were diluted to an OD_{560} of 0.05 with fresh S7 medium, containing traces of Xyl (0.005%), and cells were grown at 37 ºC with shaking. Every hour, the OD_{560} was measured. At OD_{560} = 0.2 IPTG (2 mM) was added (denoted by a pointing arrow), and the cultures were incubated for a total time of 720 min.

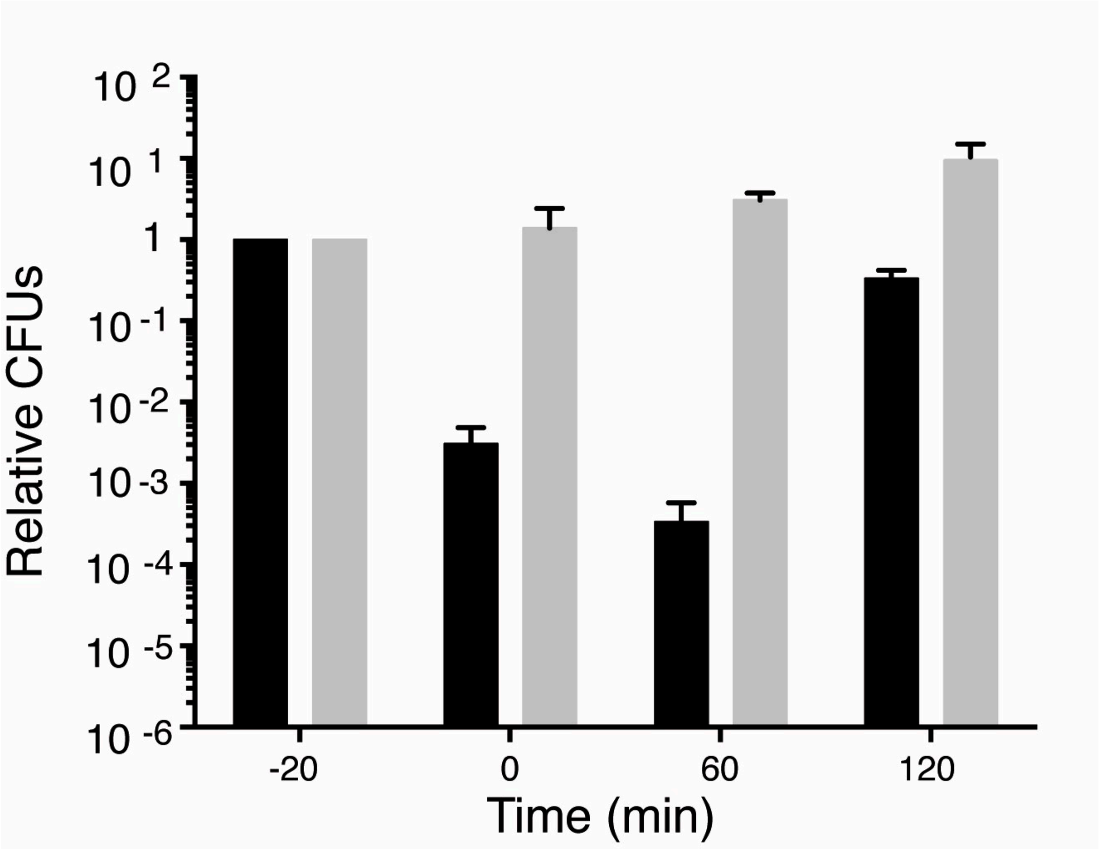

Figure S3. BG1125 (pCB799) in the presence or the absence of IPTG. BG1125 (pCB799) cells were grown in S7 medium, containing traces of Xyl (0.005%) and 10 mM $MgCl_2$ at 37 °C with shaking. Then, IPTG (2 mM) was added to half of the culture to induce z expression (time -20 min, black bar). At time 0, 60 and 120 min, samples from the culture containing (black bar) or lacking IPTG (grey bar) were withdrawn and plated in LB agar plates lacking IPTG to count the CFUs. Data are shown as mean ± standard error of the mean (SEM), from >3 independent experiments.
